# On the Classification and Reporting of Prolonged Grief: Assessment and Research Guidelines

**DOI:** 10.1097/HRP.0000000000000389

**Published:** 2024-01-05

**Authors:** Margaret S. Stroebe, Henk A. W. Schut, Maarten C. Eisma

**Affiliations:** **From** the Department of Clinical Psychology, Utrecht University (Drs. Stroebe and Schut); Department of Clinical Psychology & Experimental Psychopathology, University of Groningen (Drs. Stroebe and Eisma).

**Keywords:** bereavement, Diagnostic and Statistical Manual of Mental Disorders, Fifth Edition, Text Revision, International Classification of Diseases, 11th Revision, prevalence, prolonged grief disorder

## Abstract

**Learning Objectives: After participating in this CME activity, the psychiatrist should be better able to:**

• Explain the steps required for diagnosis of mental disorders in diagnostic handbooks.

• Identify current procedures for classifying and reporting prolonged grief disorder.

**Abstract:**

Prolonged grief disorder (PGD) was added to the 11th edition of the *International Classification of Diseases* in 2018 and to the fifth edition of the *Diagnostic and Statistical Manual of Mental Disorders* in its 2022 text revision. Thus, reporting and classifying PGD according to established guidelines has become fundamental for scientific research and clinical practice. Yet, PGD assessment instruments and criteria are still being developed and debated. The purpose of this article is to examine the adequacy of current procedures for classifying and reporting PGD in research and to suggest guidelines for future investigation and dissemination of knowledge. We outline the standard steps required for diagnosis and assessment of a mental disorder (notably, the administration of clinical interviews). In order to illustrate reporting about the presence/prevalence of PGD in recent scientific articles, we conducted a search of Scopus that identified 22 relevant articles published between 2019 and 2023. Our review of the literature shows that standard classification procedures are not (yet) followed. Prevalences of PGD are based on self-reported symptomatology, with rates derived from percentages of bereaved persons reaching a certain cutoff score on a questionnaire, without clinical interviewing. This likely results in systematic overestimation of prevalences. Nevertheless, the actual establishment of PGD prevalence was often stated in titles, abstracts, and results sections of articles. Further, the need for structured clinical interviews for diagnostic classification was frequently mentioned only among limitations in discussion sections—but was not highlighted. We conclude by providing guidelines for researching and reporting self-reported prolonged grief symptoms and the presence/prevalence of PGD.

## INTRODUCTION

The term *prolonged grief*, adopted in the more recent scientific literature and in diagnostic manuals to describe disturbed grief, has largely replaced the earlier, much-used term *complicated grief*, which encompassed chronic, inhibited, delayed, and absent grief.^1,2^ Prolonged grief has been described as grief reactions that become abnormally persistent and cause significant impairment in daily functioning.^3^ Prolonged grief has been shown to be related to, but distinguishable from, symptoms of neighboring disorders of depression and posttraumatic stress disorder, to predict lower quality of life and suicidal tendencies, and to be treated most effectively with grief-specific interventions.^4^ Accordingly, a disorder characterized by such grief reactions was added to the 11th revision of the *International Classification of Diseases* (ICD-11) as prolonged grief disorder (PGD).^5^ In 2022, a related but distinct disorder with the same name was included in the revised fifth edition of the *Diagnostic and Statistical Manual of Mental Disorders* (DSM-5-TR).^6^

Classification of prolonged grief in these diagnostic systems has meant that formal procedures to establish its presence will become mandatory in clinical practice. Developing such procedures is a multifaceted and complex process. For example, across both conceptualizations PGD is characterized by severe and persistent yearning and cognitive preoccupation with the deceased, yet the number and content of additional symptoms, diagnostic algorithms, and timing criteria differ. Consequently, few empirical comparisons have been published, and matters of validity and reliability are still debated.^6–11^

Since the two types of PGD described in the above-mentioned diagnostic systems also have unique features compared to past proposed prolonged grief conceptualizations, researchers have advocated the need for the development and use of standardized assessment tools and valid structured clinical interviews to assess these new constructs.^3,12,13^ Some new self-report screening tools to assess PGD per ICD-11 and DSM-5-TR have been developed and validated, such as the Traumatic Grief Inventory–Self Report Plus (TGI-SR+) and the International Prolonged Grief Disorder Scale (IPGDS).^14,15^ The application of such screening instruments offers a cost-effective, time-efficient method to identify those people at risk of developing grief complications, and the instruments are, as such, useful tools in survey research.

To the best of our knowledge, however, examinations of scientific procedures for classification and reporting of prolonged grief have been lacking. Of central importance are questions about the characteristics of PGD and its prevalence—that is, whether reports are accurate and whether the procedures on which researchers arrive at their conclusions are correct. If not, can guidelines be developed to improve the standard of inquiry? Scientific information on such issues can influence care provision for bereaved persons by, for example, affecting assessment and treatment of grief complications. It also affects such matters as policymaking in societies more generally—for example, through insurance coverage or provision of research funding.

The purpose of this article is to elucidate the adequacy of procedures for classifying and reporting prolonged grief in scientific articles and to suggest principles for future scientific investigation and the dissemination of knowledge. Prior to doing so, it is important to outline the standard steps in assessment required for diagnosis of mental disorders in diagnostic handbooks.

In order to ascertain diagnostic status for a mental disorder in the ICD and DSM systems, one needs not only to examine whether a person’s symptom level meets established criteria but also to employ clinical assessment; clinical judgement is needed to reach a diagnosis. To illustrate, the DSM’s fifth edition specifies that “Clinical training and experience are needed to use DSM for determining a clinical diagnosis”^6^^(p 94),^^16^^(p 5)^ and that “Diagnostic criteria are offered as guidelines for making diagnoses, and their use should be informed by clinical judgment.”^6^^(p 113),^^16^^(p^ ^22)^

In practice, establishing diagnoses would typically involve clinical interviewing—a personal exchange between clinician and client designed to systematically gather information.^17^ Clinical interviewing would involve eliciting a combination of social, medical, education, familial, psychological, developmental, and other information from the client and sometimes also from informants who know the client well (e.g., parent, spouse). Such information is often combined with comprehensive psychological assessment, including self-report questionnaire material; self-reported grief reactions are an integral part of making a clinical diagnosis. In line with these common procedures, Maj^18^ emphasized that the correct use of criteria requires considerable clinical experience and knowledge of psychopathology, and that these diagnostic indicators are not to be used in a “cookbook fashion.” For example, the DSM has long specified that clinical judgment must be exercised by professionals with “specialized clinical training.”^19^ Horowitz^20^^(pp 87–88)^ argued that the diagnosis of grief complications should be left to clinicians

who can observe the patients’ self-report scales, as well as conduct salient interviews to explore and weigh reports of subjective experience . . . The clinician is in the best position to compute the fuzzy logic of deciding whether a person in the midst of emotional extremes is on a route through the dark valley of mourning, and on a route where an adaptive grief process is sufficiently derailed and complicated to call for diagnosis of a disorder.

Thus, criteria of mental disorders alone do not provide sufficient basis for formal diagnostic categorization; much less do self-reported symptoms on a scale. It should be noted that neither the ICD nor the DSM is a diagnostic instrument; they are “simply” classification systems, providing lists of key symptoms for any particular disorder, with ICD (explicitly stating *Classification*) clearer about this than DSM (a *Diagnostic* system). It follows that diagnosis of disorder cannot be made on the basis either of reaching a cutoff point on a self-report measure or scoring high on particular items designed to screen for symptoms according to DSM/ICD: clinical interviews and judgment are imperative and serve to provide additional information to that provided in a questionnaire for developing a fuller understanding of the bereaved person’s experience. For example, DSM-5-TR notes that among associated features of PGD the clinician may need to consider are maladaptive cognitions, somatic complaints, and harmful health behaviors.^6^^(p 324)^

With the above in mind, we aimed to conduct a review to summarize reports about the presence/prevalence of PGD in recent scientific articles and describe the bases on which researchers have drawn their conclusions. We examine whether researchers follow the recommended steps outlined in the DSM and ICD diagnostic manuals for establishing a diagnosis. When they have not done so, we examine whether they have included cautionary remarks about interpreting provisional classification and any given prevalences based on self-report scales in relevant sections of selected studies. Based on the results from our review, we highlight potential risks of current trends in scientific reporting on PGD presence and prevalence. We also provide suggestions for future investigation and guidelines for researching and reporting on this topic.

## METHODS

### Search Strategy, Inclusion Criteria, and Study Selection

We searched Scopus on 14 March 2023 using the following terms: prolonged grief OR complicated grief OR persistent complex bereavement disorder OR PGD OR PCBD in the title, abstract, or keywords, AND prevalence in the title of the article. We searched only one database because we sought to draw a representative sample of the literature to examine current reporting trends, rather than providing a comprehensive review on the topic. We chose Scopus because it is a respected, comprehensive scientific database and includes more keywords for selected articles, allowing for easier identification of relevant studies (see https://faseb.onlinelibrary.wiley.com/doi/10.1096/fj.07-9492LSF). We limited our search to the years 2019–23 because we were interested in reporting on grief disorder presence/prevalence since the introduction of PGD in diagnostic handbooks. We chose to include studies that (1) were peer-reviewed English-language articles, thereby ensuring the comprehensibility and quality of included work, and (2) reported on reviews or empirical research on the prevalence of grief disorders. We initially identified 32 articles. Of those articles, 8 were excluded because they were not focused specifically on prevalence of grief disorders, 1 was excluded because it was a letter to the editor, and 1 because the article could not be retrieved, leaving 22 articles for data extraction. See Table 1.^21–42^

**Table 1:** Review of Studies on the Prevalence of Disordered Grief, 2019–2023

Study	Title of article Empirical study/review	Bases for prevalence/diagnostic reporting Gives a percentage prevalence	Qualifying remarks regarding prevalence, diagnosis from subjective self-report questionnaires (firm assumptions vs. cautious estimates), suggestive prevalence^a^	Transparency: Clear about *suggestive-only prevalence* 1. In title/abstract 2. In discussion
Boelen et al. (2019)^21^	“Further Evaluation of the Factor Structure, Prevalence, and Concurrent Validity of DSM-5 Criteria for Persistent Complex Bereavement Disorder and ICD-11 Citeria for Prolonged Grief Disorder” Empirical	Self-report ICG-R and SCL-90 depression scale PCBD symptoms (all but 1) were covered by 12 ICG-R and 3 SCL items All 12 ICD-11 PGD symptoms were covered by 9 ICG-R and 3 SCL items % prevalence reported	Cautious throughout Abstract: “The prevalence of probable DSM-5 PCBD (8.2%) was significantly lower than ICD-11 PGD (19.2%)” (p 206) and “This study was limited by its reliance on self-reported data and grief symptoms were derived from two scales” (p 206). Results and Discussion: “Probability” of caseness is sometimes mentioned. In latter, absence of interviews is explicitly mentioned as a limitation: “Symptoms were assessed using self-report measures and obtained from two different questionnaires. Future work should preferably use clinical interview-based assessment of symptoms” (p 209).	1. Yes 2. Yes
Steil et al. (2019)^22^	“Prevalence of Prolonged Grief Disorder in a Sample of Female Refugees” Empirical	Self-report participants were interviewed using the PG-13 % prevalence reported	Firm throughout Abstract: under Objectives, “examines the prevalence of PGD” (p 1); “9.41% met criteria for PGD” (p 1); Conclusion in Abstract: “The PGD prevalence rate found . . .” (p 1). Discussion: The PG-13 described as a “rigorous diagnostic tool” (p 8) and noted the criteria might in future adopt ICD-11 criteria instead of those in Prigerson et al. (2009).^23^ Final Conclusion: open to improvements but not referring to need for clinical interview for diagnostic assessment.	1. No 2. No
Zordan et al. (2019)^24^	“Long-Term Prevalence and Predictors of Prolonged Grief Disorder Amongst Bereaved Cancer Caregivers: A Cohort Study” Empirical	Self-report PG-13 % prevalence reported	Firm statements throughout Abstract: firmly stated aim to evaluate the prevalence of PGD. Discussion: does mention a need to develop a screening process for PG, though in the context of preventing PGD before it is established. No mention of need for clinical interview. At 37 months, 5% met criteria for PGD, and 14% for subthreshold PGD (categorized based on an algorithm developed by the scale authors: non-cases, subthreshold, and cases).	1. No 2. No
Djelantik et al. (2020)^25^	“The Prevalence of Prolonged Grief Disorder in Bereaved Individuals Following Unnatural Losses: Systematic Review and Meta Regression Analysis” Review	Prevalences based on self-report symptom questionnaires Covering self-report scales: ICG, ICG-R, PG-13 (see their Table 2) % prevalence reported	Firm statements in Title/Abstract; some caution in Discussion Abstract: aim to establish “pooled prevalence of PGD”; concludes “nearly half of the bereaved adults [following unnatural losses] experienced PGD” (p 146). Gives one reason for interpreting findings with caution: due to heterogeneity of study methodology. Results: mentions “PGD cases” (p 148). Discussion: “in most included studies the prevalence rates were based on self-report questionnaires. Scoring above a clinical cut-off of a self-report questionnaire should be recognized as an indication of disorder. A structured clinical interview is needed for formal diagnosis” (p 154). Also mentions need for “a standardized assessment tool for PGD” (p 154). General conclusion: “Globally, approximately half of the bereaved individuals might develop symptoms which meet the diagnostic criteria for PGD following unnatural losses” (p 155).	1. No 2. Yes
Kokou-Kpolou et al. (2020)^26^	“A Comparison of Prevalence and Risk Factor Profiles of Prolonged Grief Disorder Among French and Togolese Bereaved Adults” Empirical	Self-report: PGS-11 % prevalence reported	Firm statements in Title/Abstract; some caution in Discussion Careful formulation in the Methods: “Higher summed PGD scores (range 1–55) provide an index of the severity of potentially problematic Grief” and of “prevalence rates of probable PGD caseness” (p 759). Results: some caution, too: “probable PGD diagnosis” (p 760). Discussion: need for a clinical interview is addressed: “Other limitations were that the results are based on a cross-sectional design, self-reported data, and no clinical assessment interviews were conducted. In the future, a prospective longitudinal design paired with clinician ratings for grief symptoms would be of value” (p 762).	1. No 2. Yes
Morowatisharifabad et al. (2020)^27^	“Prevalence of Complicated Grief and Related Factors in Elderly Individuals in Sabzevar City, Iran” Empirical	Self-report: BGQ % prevalence reported	Firm statements throughout Abstract: “The prevalence of CG among the participants who had experienced grief was 18.6%” (p 718). Discussion: a disclaimer mentioned: “No precise diagnostic criteria exist for diagnosing and measuring CG, so the prevalence can vary depending on the applied diagnostic criteria” (pp 722–23). No mention of limitations by relying solely on self-report.	1. No 2. No
Zhou et al. (2020)^28^	“Prevalence and Associated Factors of Prolonged Grief Disorder in Chinese Parents Bereaved by Losing Their Only Child” Empirical	Self-report using ICD-11 PGD guidelines (see their Table 1, p 30) but selected 9 of their 10 items from PG-13 Prevalences based on self-report symptom questionnaire % prevalence reported	Firm statements, in Title and Abstract; some caution in text Methods: “qualify for a ICD-11 PGD diagnosis” (p 12). Discussion: mention of PGD symptoms, “clinically relevant diagnosis” (p 16), and prevalence as estimated (prevalence reported among these elderly Shidu parents was 13.8%). Emphasizes the use of ICD-11 guidelines, though these are all but one PG-13 items, and prevalence is based on criteria sets, not clinical judgment. It is mentioned (as second limitation in Discussion) that the study adopted “self-reported items to examine the prevalence of PGD, and future research is needed to use a structured clinical interview [to] confirm PGD caseness and explore the prevalence of PGD” (pp 19–20).	1. No 2. Yes
Bryant et al. (2021)^29^	“A Prevalence Assessment of Prolonged Grief Disorder in Syrian Refugees” Empirical	5-item previously used interview assessing PGD (consistent with ICD-11 symptoms): a (brief) self-report questionnaire in interview format % prevalence reported	Firm throughout Statements about prevalence without qualifying remarks, in main text (article has no sections): “Among those experiencing bereavement, 85 (15.1%) met criteria for PGD, which comprised 8.9% of the entire sample . . . These findings are significant as this is the first study reporting on the prevalence of PGD, by representative sampling, in a population of refugees directly affected by war” (p 302).	1. No 2. No
Djelantik et al. (2021)^30^	“Prolonged Grief Disorder, Posttraumatic Stress Disorder, and Depression Following Traffic Accidents Among Bereaved Balinese Family Members: Prevalence, Latent Classes and Cultural Correlates” Empirical	Self-report: TGI-SR % prevalence reported	Firm statements in Title/Abstract; some caution in Discussion Abstract: “Prevalence rates of PGD (0%), PTSD (1%) and moderate depression (2%) were low” (p 773). Results: slightly more caution. “None of the participants was found to suffer from a probable diagnosis of PGD” (p 776). Discussion: “probable diagnosis” is mentioned, with note of limitation that “only a structured clinical interview assessing all criteria and taking into account an examination by licensed clinicians is needed to obtain formal diagnoses” (p 779).	1. No 2. Yes
Lundorff et al. (2021)^31^	“Time Elapsed Since Loss or Grief Persistency? Prevalence and Predictors of ICD-11 Prolonged Grief Disorder Using Different Applications of the Duration Criterion” Empirical	PG-13 & ICG-R Prevalences based on self-report symptom questionnaire % prevalence reported	Firm statements in the Title; some caution in Abstract, Discussion, etc. Abstract: “Without a structured clinical interview, only probable cases of PGD were identified” (p 89). In Discussion, as justification: “as no available tool reflect[s] the current diagnostic criteria for ICD-11 PGD, the disorder was assessed by matching diagnostic symptoms with items from a confined number of questionnaires,” (p 96) implying that, had there been a valid tool, this would suffice to establish diagnostic disordered status. But they do add in Discussion that “reliance on self-report data and no structured clinical interview restricts findings to probable PGD caseness. Clinical implications should be interpreted cautiously” (p 96). Probable prevalence decreased from 18.9% at 6 months to 13.4% at 11 months post-loss.	1. Yes 2. Yes
Pan & Liu (2021)^32^	“The Prevalence of Complicated Grief Among Chinese People at High Risk: A Systematic Review and Meta-analysis” Review	Self-report: mostly PG-13 & ICG Prevalences based on symptom questionnaire % prevalence reported	Firm statements throughout Abstract: they reviewed “the rate of CG among Chinese people” (p 480), covering studies that calculate prevalences of CG in Chinese settings. Results: reference to “the tools for diagnosing CG” (p 485), with the tools being the established scales. In discussion, while critical of the database, the authors infer the “pooled prevalence of CG” (p 487), with the take-home message that they are actually exploring the rates of a diagnostic entity. Pooled prevalence estimate of CG was 22.0%.	1. No 2. No (see column immediately to left)
Parro-Jiménez et al. (2021)^33^	“Complicated Grief: A Systematic Review of Prevalence, Diagnosis, Risk and Protective Factors in the Adult Population of Spain” Review	Self-report using a range of assessments and different “detection instruments” (covering various “diagnostic instruments” and self-report symptom questionnaires (e.g., ICG, TRIG) % prevalence reported	Cautious throughout Abstract: noted prevalences based on scales such as the ICG, but cautious and discriminating (see column 3 here), though acknowledged usefulness of symptomatic measuring instruments. In Discussion: concluded that prevalence rates may be overestimated: “they usually yield higher data because there is a simple and direct correspondence between the scale score and the presence and severity of the disorder. On the contrary, the polythetic criterion of diagnostic classifications is more demanding, as it makes it necessary to present a set of symptoms, under specific temporal and severity criteria, in order to refer to a disorder” (p 197). Prevalence: weighted mean prevalence (based on 6 of the reviewed studies) was 21.56%; 7.67%–10.68% in those using diagnostic, and 28.7% in those using symptomatic, instruments.	1. Yes 2. Yes
Rosner et al. (2021)^34^	“Prevalence of Prolonged Grief Disorder” Empirical	Self-report based on extended version of the self-reported PGD Scale (PG13 + 9) % prevalence reported	Cautious throughout Abstract: “Results are based on self-reported data. The PG13 + 9 was not designed to assess grief symptoms according to ICD-11 and DSM-5-TR diagnostic criteria” (p 301), and reports there “the probable prevalence of PGD” (p 301). Among bereaved samples the prevalence was 4.2% (ICD-11); 3.3% (DSM-5-TR). Results (in Abstract) “are based on self-reported data. The PG13 + 9 was not designed to assess grief symptoms according to ICD-11 and DSM-5-TR diagnostic criteria” (p 301). Discussion: further caution under Limitations: “although diagnostic status of prolonged grief was assessed using ICD-11 and DSM5–TR classification rules, our results are based on self-reported data, not on clinician-administered structured interviews” (p 306).	1. Yes 2. Yes
Tang & Xiang (2021)^35^	“Who Suffered Most After Deaths Due to COVID-19? Prevalence and Correlates of Prolonged Grief Disorder in COVID-19 Related Bereaved Adults” Empirical	Self-report using 13-item International PGD Scale (IPGDS: 1–65) and 17- item TGI-SR (TGI-SR: 1–85) % prevalence reported	Cautious throughout Abstract: “aimed to *estimate* [emphasis added] the prevalence of PGD, and mentioned prolonged grief symptoms” (p 1), not diagnosis. Results: notes that prevalence was screened by two self-report symptom scales (37.8% screened by International PGD Scale; 29.3% by Traumatic Grief Inventory Self Report). Discussion: Uses “the latest diagnostic criteria of pathological grief in ICD-11 and DSM-5 and the most updated measures for PGD and PCBD” (p 5), so quite transparent, but claims that the rates show prevalence of individuals suffering from PGD or PCBD. Discussion, in limitations: “although strictly following the ICD-11 and DSM-5 diagnostic guidelines, this study adopted self-reported rather than structured clinical interviews to determine prevalence rates” (pp 7–8).	1. Yes 2. Yes
Baumann et al. (2022)^36^	“Prolonged Grief, Posttraumatic Stress, and Depression Among Bereaved Parents: Prevalence and Response to an Intervention Program” Empirical	Self-report: ICG-D (German version of ICG) for CG, with added self-construed items to cover ICD-11 PGD criteria % prevalence reported	Cautious throughout; clear that scores are only suggestive Abstract: “The baseline assessments indicated that 160 (49.5%) parents showed symptoms of prolonged grief disorder (PGD). Complicated grief was indicated in 272 (84.2%)” (p 837). Methods: “An endorsement of 3 (often) or 4 (always) was assumed to indicate clinically relevant symptoms” (p 842). Results: “49.5% of participants showed severe symptoms of PGD on admission to the program. Clinically relevant complicated grief was indicated in 84.2% of the participants” (p 846). In the table: “Likely to meet diagnostic criteria” (p 848) is added when presenting numbers and percentages. Discussion: explicit cautionary statement: “Furthermore, all data processed for this study are based on self-report questionnaires, and no formal clinical assessments were utilized. Therefore, the prevalence of mental disorders was estimated solely on the basis of self-report questionnaire data” (p 850).	1. Yes 2. Yes
Işikli et al. (2022)^37^	“Validation of the Prolonged Grief Scale (PG-13) and Investigation of the Prevalence and Risk Factors of Prolonged Grief Disorder in Turkish Bereaved Samples” Empirical	Self-report: PG-13 translated into Turkish % prevalence reported	Firm statements in Title/Abstract; some caution in Discussion Abstract: “PGD prevalence rates” and “risk factors for the PGD diagnosis” (p 628). Results: “the prevalence of PGD was [11.4% in study 1; 10% in study 2]” (p 634). Discussion: “the PG-13 is a reliable and valid measurement tool that can be used to measure PGD” (p 637), and “Self-reported measurements were used instead of a structured clinical interview” (p 637), without indication that self-reporting is only approximate. Does mention that PGD criteria are not identical with ICD-11 or DSM-5 criteria and that results may be different with other diagnostic criteria.	1. No 2. Yes
Titlestad & Dyregrov (2022)^38^	“Does ‘Time Heal All Wounds?’ The Prevalence and Predictors of Prolonged Grief Among Drug-Death Bereaved” Empirical	Self-report: PG-13 and a Special Grief Questions measure % prevalence reported	Cautious throughout Abstract: “The study aimed to estimate the prevalence of prolonged grief (PG) symptoms” (p 1). Methods: Although prevalence is calculated, it is for PG symptom levels reaching “a preliminary PG cut-off score” (pp 7–8), not diagnosis/PGD. “PG symptoms” emphasized throughout. Discussion: “The outcome measure PG-13 is a standardized questionnaire that is used by clinicians to diagnose prolonged grief disorder in bereaved people. We have therefore not made assumptions about a disorder, but rather, have described levels and prevalence of prolonged grief symptoms referring to sum-scores from the instrument” (pp 16–17). Prevalence above tentative cutoff scores for PGD = 26%.	1. Yes 2. Yes
Titlestad et al. (2022)^39^	“Prevalence and Predictors of Prolonged Grief Symptoms Among Those Bereaved from a Drug-Related Death in a Convenience Sample of Norwegian Parents: A Cross-sectional Study” Empirical	Self-report: PG-13 & a Special Grief Questions measure Prevalence is given in terms of PG symptom level (means), not disorder/diagnosis % prevalence not reported	Cautious throughout (involving a subsample from the END study, with a focus on symptoms not diagnosis/disorder [see Titlestad & Dyregrov (2022)^38^]) Introduction: purpose of study included “to map the prevalence of PG symptoms” (p 1356). Discussion: specified that it was to “identify predictors of symptoms” (p 1359), not disorder prevalence. Methods: “There is no official cut-off score, but a preliminary cut-off score of 35 or more [has been suggested], which meets the diagnostic criteria for PGD” (p 1357). Discussion: similar cautions to those of Titlestad & Dyregrov (2022):^38^ “PG-13 is a diagnostic tool used by clinicians in structured clinical interviews; however, we used it to collect self-reported data. Assumptions relating to meeting criteria for the disorders should, therefore, not be made” (p 1361).	1. Yes 2. Yes
Treml et al. (2022)^40^	“Prevalence, Factor Structure and Correlates of DSM-5-TR Criteria for Prolonged Grief Disorder” Empirical	Self-report using items from German version of PG-13 and ICG % prevalence reported	Firm in Abstract, cautious in text Abstract: although stated (also in Introduction) that a PGD *estimate* is used, no other indication is given there or in the title that clinical interviews are necessary. Also, mentions that the prevalence is derived by “using the new diagnostic criteria” (p 1) (items could be matched to DSM-5-TR criteria for PGD); mentions PGD “caseness” (e.g., p 5), and “conditional” prevalence of PGD using the DSM-5-TR diagnostic algorithm (p 6). Conditional prevalence was 3.4%. Discussion: notes aim to “explore the prevalence rate of the new DSM-5-TR criteria for PGD” (p 6). Acknowledges the limitation that the “results are based on self-reported questionnaires rather than clinician-administered structured interviews. The exclusive use of self-report measures could have evoked a bias due to misinterpretation of questions” (p 8). Thus, the limitation is attributed more to a possibility that respondents’ answers may be biased rather than that clinical interviews are an essential component of the diagnostic procedure.	1. No 2. Yes
Wilson et al. (2022)^41^	“What Exactly Is ‘Complicated’ Grief? A Scoping Research Literature Review to Understand Its Risk Factors and Prevalence” Review	Self-report: ICG, PG-13, and other tools % prevalence reported	Firm statements throughout Abstract (and in the text): focus on diagnosis, not cautionary, no disclaiming (e.g., mentioning the 6-month timeframe for diagnosis in Abstract), reference to “diagnostic tests” (p 471). Discussion: calling for practical measures such as reports by family members/friends or responding when someone asks for help after 6 months. No mention of clinical interviewing. Considerably varying incidence/prevalence rates from 8.2% to 62.8%—the latter in connection with a suicide bereavement center.	1. No 2. No
Yuan et al. (2022)^42^	“Prevalence of Prolonged Grief Disorder and Its Symptoms in Chinese Parents Who Lost Their Only Child: A Systematic Review and Meta-analysis” Review	Self-report: PG-13, ICG % prevalence reported	Firm statements throughout Abstract and text: reports prevalences of PGD (20.9%) and PGD symptoms (75.0%) (their review said to cover studies of both, as detailed in Results). Firm conclusion in Abstract that “PGD is prevalent” among these parents. Results: 5 self-reports, 2 in-person interviews (neither apparently clinical interviews). One used the PG-13; the other was Zhou et al. (2020)^28^ (see above). Since 6 of the studies were described as focusing on PGD, prevalences of diagnosis *include those reported in the 5 self-report studies*, thus suggesting valid PGD assessment through those self-reports. Discussion: refers to the “true prevalence of PGD” (p 7) (which may have been overestimated—implying that PGD has been measured and that self-reporting is sufficient).	1. No 2. No
Thiemann et al. (2023)^43^	“Prolonged Grief Disorder Prevalence in Adults 65 Years and Over: A Systematic Review” Review	Self-report, with “use of varying PGD-constructs which did not match ICD-11 and DSM, Fifth Edition criteria” (Abstract): ICG, ICG-R, and PG-13 (see their Tables 2 & 3) Prevalences based on self-report symptom questionnaire (?) % prevalence reported	Firm in Abstract; at times firm and other times cautious in the text Abstract: “a similar proportion of older adults experience PGD as the general bereaved adult population,” (p 1) but results are also presented here as the “*Conditional* [emphasis added] prevalence for PGD” (p 1), implying that “disorder” has been assessed. Conclusion: mentions as a limitation “shortcomings in . . . [PGD] assessment including the use of self-report measures only and measures not accounting for all criteria” (p 10). But here, too: clinical interviewing is mentioned as an *aid to accuracy* rather than as a prognostic requirement: “To help evaluate the current evidence[,] performance of self-reports for PGD should be evaluated against clinical interviews” (p 10). Criticized the use of varying PGD constructs that did not match ICD-11 or DSM-5 criteria. Conditional prevalence of PGD ranged from 3.2%–48.8%.	1. No 2. Yes

BGQ, Brief Grief Questionnaire; CG, complicated grief; ICG, Inventory of Complicated Grief;^44^ ICG-R, Inventory of Complicated Grief–Revised;^45^ ITG, Inventory of Traumatic Grief; PCBD, persistent complex bereavement disorder; PGD, prolonged grief disorder; PG-13, Prolonged Grief Disorder Scale;^23^ PGS-11, Prolonged Grief Scale; SCL, Symptom Checklist; TGI-SR, Traumatic Grief Inventory Self-Report;^46,47^ TRIG, Texas Revised Inventory of Grief.^48^

^a^ Use of the term *firm* refers to instances in which assumptions about diagnostic prevalences are made based on survey self-report data. *Cautious* refers to statements indicating that prevalences derived from survey self-report data are estimates, suggestive of diagnosis but not based on clinical interview.

### Data Extraction

The first and second authors (MSS and HAWS) extracted the following data: authors and publication year, article title, instruments/procedure on the basis which prevalence or presence of prolonged grief was established, and qualifying remarks on prevalence or (provisional) diagnoses in the title, abstract, methods, results, and discussion sections. Specifically for (a) the title and abstract and (b) the discussion, we coded whether authors were transparent (how clear they were about the prevalence being only suggestive, not diagnostic) in their reporting of the extent to which they could make statements about prevalences and diagnoses of prolonged grief (answer options: Yes, No). An affirmative coding was made if authors stated explicitly that the prevalence was only an estimate based on self-report questionnaire data. A negative coding was assigned if assumptions of diagnostic status were made from self-reported questionnaire data. This information is listed for each article in the qualifying remarks in Table 1, column 4.

## RESULTS

### Study Characteristics, Assessment Instruments, and Reports About the Presence/Prevalence of Prolonged Grief

Sixteen included articles were empirical studies and six were reviews. Twenty-one included articles either reported on empirical research using self-report scales (including versions of the Inventory of Complicated Grief [ICG],^44,45^ Prolonged Grief 13 Scale [PG-13],^23^ and Traumatic Grief Inventory–Self Report [TGI-SR])^46,47^ or reviewed information primarily obtained from these scales. One study derived five items from a previously developed interview to approximate PGD ICD-11 criteria, but was not specifically designed to assess this criteria set; as such, the process could not be considered a formal clinical interview.^29^ No articles used recommended clinical procedures—namely, clinical interviews—to establish diagnoses. Twenty-one articles reported prevalences. As shown in Table 1 (column 4), estimated prevalences varied widely. This diversity related, for example, to the duration of bereavement, to the particular sample of bereaved participants, or to the measure or cutoff point used (see also Kokou-Kpolou et al. [2020]^26^ and Lundorff et al. [2021]^31^). One study derived prevalence estimates by PG symptom levels rather than by provisionally classifying people as having or not having a disorder.^39^

### Cautionary Statements About the Prevalence of Prolonged Grief Based on Self-Report Scales

Table 1 (column 4) gives detailed information about the extent to which each article includes cautionary statements regarding the prevalence of prolonged grief based on only the self-report information. For clarity (see also Table 1, footnote a): (1) We use the term *firm* in these descriptions when survey self-report data have been considered to determine diagnostic prevalences. (2) By contrast, *cautious* refers to statements indicating that survey self-reports indicate an *estimation*, potentially suggestive of diagnosis but not based on the necessary clinical interview procedures.

As summarized in Table 1, only eight of the listed articles (37%) explicitly highlighted—both in their titles/abstracts and discussion sections—the approximate, limited nature of self-report ratings in determining PGD prevalences and the need for formal clinical assessment as integral to establishing diagnoses. Seven of the listed articles (32%) contained cautionary statements on this issue only in the limitation section of the discussion, and seven (32%) did not contain any cautionary statements.

As the details included in Table 1, column 4, indicate, for the 15 articles that did include some form of cautionary statement, the extent to which they did so varied considerably, even among those classified as expressing caution in both the title/abstract and discussion. As shown in descriptive details in Table 1, some authors make this limitation very evident in both sections. For example, two articles explicitly mention the lack of formal clinical assessments as a limitation precluding firm conclusions about classification of PGD.^21,36^ Others that we have counted as “cautious” are actually less so. For example, one article was included in this category because the use/value of symptomatic versus diagnostic instruments was compared, though the need for clinical interviewing to establish diagnoses was not detailed.^33^ In other articles, while the word *conditional* was used to describe prevalences, further explanation was lacking, and the article was thus classified as not providing qualifying statements (see, e.g., Treml et al. [2022]^40^ and Thiemann et al. [2023]^43^).

## DISCUSSION

Since PGD was only recently added to the ICD-11 and DSM-5 systems, time is needed for the field to develop, to validate diagnostic criteria, and to refine procedures. This article aims to facilitate this process by investigating a limitation of current scientific work and suggesting guidelines for improvement. Our review of the scientific literature has shown that, so far, reports about the presence of prolonged grief among bereaved individuals and about prevalences among (sub)populations rely heavily on self-report measures. Self-report instruments to assess levels of symptoms—first among them the Inventory of Complicated Grief (ICG)^44^—have been applied to indicate (in the sense of *establish*) diagnostic status. These questionnaires have become a central feature of this general endeavor. Our review also demonstrates that mentioning the limitations of using self-report data in this manner and acknowledging the need to use recommended diagnostic approaches to establish disorder are rarely explicitly acknowledged in all relevant parts of any particular article.

While outside the time interval of our literature search, the use of questionnaire measures to determine diagnostic status, along with the need for caution in reporting prevalences, is perhaps best illustrated by an influential meta-analytic review by Lundorff and colleagues^49^ entitled “Prevalence of Prolonged Grief Disorder in Adult Bereavement: A Systematic Review and Meta-analysis.” Information from the reviewed studies on prevalences was derived from calculating percentages of bereaved persons reaching/exceeding cutoff points on various self-report questionnaires. These questionnaires, which measured varying symptom sets, were used in this review to determine an overall estimated prevalence of 10% (see their Table 1). The authors were careful to indicate absence of use of diagnostic criteria: they conclude “one out of ten bereaved adults is *at risk* for PGD”;^49^^(p 138)^ they note that this proportion exhibited “*clinically relevant levels* of PGD symptoms”;^49^^(p 146)^ and they mention the need for applied assessment instruments to be “*combined with clinical diagnostic interviews* to secure a correct classification of PGD”^49^^(p 148)^ (emphasis added). Many of the included articles in our review included a reference to this article, using the prevalence of 10% as a “gold standard” against which to compare the prevalence of prolonged grief in their own studies. Typically, however, these studies do not appropriately acknowledge the cautions formulated in the Lundorff review.^49^

Further to the points discussed above, in our view, attention to limitations inherent in relying solely on self-reports to screen for or determine diagnostic status is needed. Narrow assessment provides limited information regarding a client’s symptom profile and functional impairment. Reliance on the client as the sole source of (self-reported) information is insufficient in the context of diagnosis and clinical decision-making. Finally, personal and societal context need to be taken into account (e.g., the availability of supportive resources; the bereaved person’s life circumstances; cultural affiliation). These factors affect not only clinical diagnosis but also other consequential clinical decisions, such as those with respect to psychoeducation and treatment priorities.

As researchers and clinicians are well aware, authors use titles and abstracts to highlight their aims and goals in writing their articles, and potential readers use those same titles and abstracts to understand the article’s main findings and to determine whether the article is worth reading. In most of the selected articles, however, the titles and abstracts are misleading about whether diagnostic status had actually been established: almost two-thirds of all articles suggest that the steps described above to diagnose prolonged grief were indeed followed, when in fact they were not. For example, titles include phrases such as

– “the prevalence of PGD in bereaved individuals”
– “prevalence and predictors of ICD-11 PGD”
– “prevalence and associated factors of PGD”

Additionally, abstracts accompanying such titles often do not contain relevant information to qualify the titles. Thereby, the articles do not fully represent the nature of the scientific basis of the authors’ investigations.

Nearly all authors proceed to give percentages of bereaved individuals suffering from prolonged grief, either in their own specific samples or in the context of a review, making firm statements in their results sections about proportions of people with grief complications.

How cautious, then, are the authors when presenting numbers on prevalence on the basis of self-reported information? As evident in Table 1, the extent to which authors indicate the insufficiency of self-report symptom levels for assessing diagnostic status and prevalence varies considerably. Some authors clearly acknowledge this limitation, whereas others give minimal recognition that symptom levels are not equivalent to diagnostic status. One-third of authors do not acknowledge this issue in any part of their articles. Of those that do, some are attentive to the difference between self-report and diagnostic assessment throughout their articles. For example, although some titles suggest that a self-report inventory is a measure of PGD, the authors report that prevalence was based on self-report scores, acknowledging that this is only an “indication” or “provisional”. Others note, when estimating prevalence, that self-report scales identify “probable” cases and that findings should be interpreted with caution. Some describe the use of symptom levels as a *proxy* for diagnostic and interview assessment of PGD, making clear that the results from self-reported symptoms do not equate a diagnosis of mental disorder.

While not the primary focus of our review, one additional problem concerns the use of the term prolonged grief *disorder* for prolonged grief disorder *symptoms* assessed with outdated scales (e.g., versions of the ICG). These outdated scales either do not actually assess the most recent criteria sets or only approximate them (for discussions of diverse assessments of PGD symptoms across instruments/diagnostic categorizations, see Treml et al. [2020],^3^ Eisma [2023],^4^ and Haneveld et al. [2022]^10^). To elaborate: very few of the scales included in the selected studies comprehensively assessed all specified symptoms of PGD per ICD-11 and DSM-5-TR. For example, the ICG and PG-13 do not completely cover current criteria sets of PGD. The measurement techniques thus often fall short of the mandatory procedure for establishing a PGD diagnosis in this respect as well. Since current PGD criteria differ from past proposed criteria sets in symptom content, count, and diagnostic algorithms,^4,10^ it cannot be assumed that their phenomenological characteristics are the same (see, e.g., American Psychiatric Association [2022]^6^ and Eisma et al. [2020]^12^). This could form an important source of bias in estimates of prevalence.

The main results of this review thus indicate that established procedures are not adequately followed when determining the presence and prevalence of prolonged grief and that the common practice of deriving such information from self-report scales is rarely appropriately specified as a limitation in scientific articles. We therefore need to ask whether, and to what extent, this shortcoming should be considered problematic.

How probable is a diagnosis of PGD based on self-report questionnaire scores? Clinical interviews to assess PGD are not yet broadly undertaken, making it difficult to provide direct evidence for any reports on the accuracy of using self-report data for classification of people with grief disorders. Relevant information on this question can be derived from research on neighboring disorders, however, such as major depressive disorder. The ease of administration offered by self-report questionnaires has made them popular tools to screen for depression in epidemiological research.^50^ Typically, cutoffs for these questionnaires are developed by comparing scores against the results of structured clinical interviews. When developing cutoffs the primary aims are to maximize the probability that a person with major depression is correctly classified (sensitivity) and the probability that a person without major depression is correctly classified (specificity).^51^ Because screening cutoffs are developed to identify all people at risk for disorder, they typically “cast a wide net,” with many people whose symptoms meet a cutoff score not actually meeting criteria for the diagnosis. In other words, they maximize sensitivity, while clinical interviews improve specificity and thereby correct potential bias toward overselection.

In many medical settings, fewer than three of ten patients whose symptoms screen positively for depression meet criteria for a depressive disorder.^52^ Similarly, meta-analyses comparing prevalences of depression based on self-report instruments and clinical interviews typically find that self-report cutoffs systematically overestimate prevalence (for a review, see Thombs et al. [2018]^50^). For example, in a meta-analysis involving patients who underwent bariatric surgery, 19% had depression in 34 studies that were based on evaluation by screening questionnaires, but the rate was 7% to 8% in 6 studies that used a validated diagnostic interview.^53^ Another review of meta-analyses showed that self-report tools estimated the prevalence of depressive disorders as being almost twice as high as the estimated prevalence based on validated clinical interviews.^54^

Notably, the cutoffs used in research on prolonged grief may be even less accurate than those used in research on depressive disorders. One of the most popular cutoffs for questionnaires for prolonged grief (a score >25 on the ICG) is not based on a comparison against results from a validated clinical interview. Recent cutoffs are based on comparisons of total scale scores against people scoring high on items tapping into a particular PGD criteria set on the same self-report instrument (i.e. a score of 4 or higher on a 5-point Likert scale).^14,44^ Therefore, the use of cutoff scores for self-report instruments for prolonged grief (compared to depression) may be even more likely to lead to systematic overestimation of the number of true cases.

What then, are the main consequences of assuming pathology from self-reported symptoms? The hope is to avoid pathologizing/overdiagnosis of normal grief reactions but also to enable those with disordered reactions to get the professional assistance they may need.^8^ Again, to cite Horowitz:

The reason to have a diagnosis for complicated grief is that there are mourning responses that do not head for mending, and that might be helped by diagnosis, formulation, and treatment covered by insurance . . . there is perhaps danger to such a diagnosis, which is a stigmatization of a normative crisis of the human condition. I believe there is a greater danger to patients: non-payment by third party managers for treatment because the patient does not receive a listed diagnosis that “requires” professional clinical help.^20^^(p 87)^

One needs to take into account the possibility that giving a diagnostic label may hurt some people, with the potential to be stigmatizing,^55^ while others may feel relief on receiving diagnosis.^56^ Taken together, such lines of reasoning point to the complex impact of assigning bereaved people to diagnostic status, and underline the argument that such diagnosis must be made as accurately as possible.

An equally difficult issue concerns the interpretation of scores above cutoff points. It is not unusual to find high proportions of subjects with such scores; for example, in a recent review the conclusion was that, following unnatural losses, nearly half of the bereaved adults experienced PGD.^30^ One concern is that the pooled prevalence was based on self-rated scores in voluntary response samples. Another is whether high scores indicate “derailment” of their grieving or whether, to some extent, the grieving may be the expected and normal, if impactful, reaction to the unnatural losses. Potentially, one comes to terms with such losses over time, without needing, or benefiting from, intervention. Some evidence suggests, however, that “indicated” interventions for at-risk bereaved persons reduce symptomatology even if their symptom levels do not strictly meet criteria for PGD (e.g., Johannsen et al. [2019],^57^ Litz et al. [2014],^58^ and Reitsma et al. [2023]^59^). Again, such considerations point to the importance of expert clinical judgment for interpreting reactions in terms of clinical significance, potential psychopathology, or impaired functioning. According to both DSM-5-TR and ICD-11, the duration and severity of distress relating to the deceased person, as well as functional impairment, have to be established as clinically significant.

### Guidelines and Directions for Future Research

A key goal of this article was to examine whether the scientific literature accurately conveys what can be correctly inferred regarding diagnostic status from survey data: do authors make clear that scores reaching cutoff points on questionnaires only suggest *potential clinical relevance* but do not indicate *diagnostic status*? The answer is no, not well enough yet. We identified recurrent shortcomings in the reviewed studies regarding the reporting of prolonged grief symptoms based on self-report data.

Given these problems and the DSM/ICD guidelines discussed above, we have developed some preliminary guidelines to help direct future reporting and research. In particular, we suggest a number of fundamental guidelines, for use in both empirical and review articles, for improving accuracy when discussing self-report, questionnaire-based estimates of prolonged grief. See Text Box 1.

**Text Box 1**

**Reporting Self-Rated Prolonged Grief Symptoms: Summary of Guidelines**

Be aware/convey these messages

– Reaching cutoff points indicates potential grief complications, not their presence.
– Prevalence rates for PGD require clinical evaluation of diagnostic status.
– Realize/acknowledge scores over a cutoff point may indicate normal reactions to “abnormal” experience.

Be explicit

– Reaching a cutoff point on a self-report questionnaire is not the same as establishing diagnostic status.
– Use “symptoms” or “grief severity,” not “diagnosis” or “disorder,” when assessment is based on self-report questionnaires.

Draw correct inferences about diagnostic status

– Reaching a cutoff score on either a questionnaire or symptoms stated in diagnostic criteria is only suggestive of diagnostic status.
– Clinical judgment, not merely cutoff scores, is needed to establish diagnostic status.

Differentiate symptom assessment from diagnostic assessment

– Specify PG symptoms, not PG disorder, in article titles.
– Use terms such as *indications*, *estimates*, *probable/possible disorder* when questionnaire cutoff scores alone are reported.

Acknowledge differences in measurement

– Past measures differ from current PGD criteria.
– When applying instruments not fully screening for current PGD criteria, report this limitation.

PG, prolonged grief; PGD, prolonged grief disorder.

First, it needs to be stated explicitly that reaching a cutoff point on a self-report questionnaire is not the same as establishing diagnostic status. Further, the terms *symptoms* or *grief severity*, not *diagnosis* or *disorder*, should be used when self-report questionnaires are the basis for assessment. Thus, correct inferences and conclusions about diagnostic status need to be made:

– Reaching a self-report symptom cutoff point on a questionnaire is only suggestive of diagnostic status.
– Scoring high on self-report symptoms stated in diagnostic criteria is also only suggestive of diagnostic status.
– Clinical judgment, not just self-report scores, is needed to establish diagnostic status, as is specifically noted in both DSM-5^16^^(pp^^5–6)^ and DSM-5-TR.^6^^(pp 94,113)^

Second, symptom assessment must be differentiated from diagnostic assessment. For example:

– The *titles* of articles should specify that they address prolonged grief *symptoms*, not prolonged grief *disorder*.
– Words such as *indications*, *estimates*, *probable/possible disorder* should be used when assessments of potential prevalence/classification are made from questionnaire cutoff scores.
– Differences between past measures and current PGD criteria should be acknowledged.
– When applying instruments that do not fully screen for current PGD criteria (ICG, PG-13, TGI-SR), transparently report this limitation.

Next, we pinpoint some lines for future research investigation. In order to assess PGD according to ICD-11^5^ and DSM-5-TR,^6^ it is essential to continue efforts directed toward developing and validating clinical diagnostic interviews. Uniformity and consensus regarding the conceptualization and measurement of prolonged grief across diagnostic systems would clearly be advantageous. A unified construct is urgently needed to best differentiate between bereaved people with and without grief complications. Questions concerning the sensitivity and specificity of assessment instruments need to be addressed: How many bereaved people scoring above cutoff points on questionnaires are classified as having a disorder following formal, clinical investigation? Does assessment solely on basis of scores above the cutoff point on self-report measures lead to an over- or underestimation of those diagnosed with prolonged grief following clinical interviews? The positive and negative predictive value of assessment instruments also needs to be established: How many reaching the cutoff point on any particular self-report questionnaire actually have PGD? How many not reaching the cutoff point on any particular self-report questionnaire actually do not have the diagnosis of PGD? Finally, researchers can usefully aim to apply instruments matching questionnaire items with diagnostic criteria, with the consequence that the measures account for all current criteria. Examples of validated screening instruments covering recent conceptualizations of PGD include the Traumatic Grief Inventory Self-Report Plus^14^ and the International Prolonged Grief Disorder Scale.^15^

## Conclusions

Our review has demonstrated major limitations in the assessment of prolonged grief. Scientific reporting of presence and prevalence rates of PGD are largely based on numbers of bereaved persons reaching cutoff points on self-report questionnaires. Professional clinical judgment has not been appropriately taken into account in assessment or scientific reporting. The procedures currently adopted do not correspond to formal recommendations for establishing diagnostic status in diagnostic handbooks.^5,6^ The likely consequence is an overestimation of the actual prevalences of prolonged grief. We have provided recommendations in Text Box 1 to (1) ensure that evaluation is based on proper use of diagnostic criteria and clinical judgment, (2) improve reporting accuracy of presence and of prevalence rates, and (3) report results with greater transparency about prevalences, especially when these are based solely on self-reported symptom scores. While our review was limited to recent research on the *prevalence* of grief disorders, we believe that our analysis equally applies to research focused on issues such as the phenomenological characteristics of PGD or the effects of interventions for people suffering from PGD.
